# Genetics of Psoriasis and Pharmacogenetics of Biological Drugs

**DOI:** 10.1155/2013/613086

**Published:** 2013-08-28

**Authors:** Rocío Prieto-Pérez, Teresa Cabaleiro, Esteban Daudén, Dolores Ochoa, Manuel Roman, Francisco Abad-Santos

**Affiliations:** ^1^Servicio de Farmacología Clínica, Hospital Universitario de la Princesa, Instituto Teófilo Hernando, Universidad Autónoma de Madrid and Instituto de Investigación Sanitaria Princesa (IP), 28006 Madrid, Spain; ^2^Servicio de Dermatología, Hospital Universitario de la Princesa, Instituto de Investigación Sanitaria Princesa (IP), 28006 Madrid, Spain; ^3^Centro de Investigación Biomédica en Red de Enfermedades Hepáticas y Digestivas (CIBERehd), Instituto de Salud Carlos III, 28006 Madrid, Spain

## Abstract

Psoriasis is a chronic inflammatory disease of the skin. The causes of psoriasis are unknown, although family and twin studies have shown genetic factors to play a key role in its development. The many genes associated with psoriasis and the immune response include *TNF**α**, IL23*, and *IL12*. Advances in knowledge of the pathogenesis of psoriasis have enabled the development of new drugs that target cytokines (e.g., etanercept, adalimumab, and infliximab, which target TNF**α**, and ustekinumab, which targets the p40 subunit of IL23 and IL12). These drugs have improved the safety and efficacy of treatment in comparison with previous therapies. However, not all patients respond equally to treatment, possibly owing to interindividual genetic variability. In this review, we describe the genes associated with psoriasis and the immune response, the biological drugs used to treat chronic severe plaque psoriasis, new drugs in phase II and III trials, and current knowledge on the implications of pharmacogenomics in predicting response to these treatments.

## 1. Introduction

Psoriasis is a chronic inflammatory disease of the skin which is characterized by the presence of erythematous scaly plaques [1]. The prevalence of psoriasis is 2-3% worldwide [2]. Psoriasis has a negative impact on the patient's health and quality of life, is associated with serious medical comorbidities, and affects the quality of life of family members [3, 4].

While the exact cause of psoriasis is unknown, genetic and environmental factors play an important role in its development [5].

The environmental factors that appear to influence the course of and the susceptibility to psoriasis include chronic infections, stress, low humidity, drugs (beta-blockers, lithium, antimalarial agents, and interferon), smoking, and obesity [6].

The role of genetics in the pathogenesis of the disease is well documented in family and twin studies [7]. Genetic factors have been well studied in candidate-driven gene-specific studies and in genomewide association studies (GWAS). The genome regions most strongly associated with the development of the disease are associated with the immune system. Interleukin 23 receptor (*IL23R*), *IL12B*, and the human leukocyte antigen Cw6 (*HLA-Cw6*) of the major histocompatibility complex have been strongly associated with psoriasis [8]. Several studies have described the important role of single-nucleotide polymorphisms (SNPs) in the promoter region of the tumour necrosis factor gene (*TNF*α**) [8].

Discovery of such consistent associations has enabled the development of new, more effective drugs with various targets, such as the p40 subunit of IL-12/23 (ustekinumab) and TNF*α* (infliximab, adalimumab, and etanercept) [1]. Other biological drugs are in phase III trials and include those targeting IL17 (ixekizumab and secukinumab) and the IL17 receptor (anti-IL17R) (brodalumab), all of which are administered subcutaneously [9]. Phase II clinical trials have demonstrated the efficacy and safety of inhibitors of Janus kinase (JAK) (tofacitinib) and phosphodiesterase 4 (PDE4) (apremilast) [3, 10–13], which are administered orally and may be less expensive than biological drugs.

Although these new drugs have improved tolerability and response to treatment, researchers must increase their knowledge of psoriasis in order to find additional options for oral treatment that are safer, more effective, and free of serious side effects. The influence of genetic polymorphisms on the response to biological drugs has been demonstrated in psoriasis [14, 15]; therefore, advances in pharmacogenetics would enable us to tailor treatment.

In this paper, we describe SNPs in genes associated with psoriasis and those associated with the immune response. We also review current knowledge on biological drugs and the impact of polymorphisms on the response to treatment of psoriasis.

## 2. Genetics of Psoriasis

The immune system plays a key role in psoriasis. Macrophage activation triggers an immune response that releases TNF*α*, IL1*β*, IL12, and IL23 [8]. Psoriasis has been associated with genes involved in the immune response, namely, *TNF*α**, *IL12B*, and *IL23R* [8]. However, there has also been associated with genes not involved in immune pathways, such as the early differentiation keratinization markers involucrin (*IVL*) and small proline-rich protein (*SPRR*). These genes are involved in atypical epidermal cellular organization and differentiation [16] and are upregulated in psoriasis [17]. A review of the genes and SNPs associated with psoriasis and the immune system is presented in Table 1.

**Figure 1:**
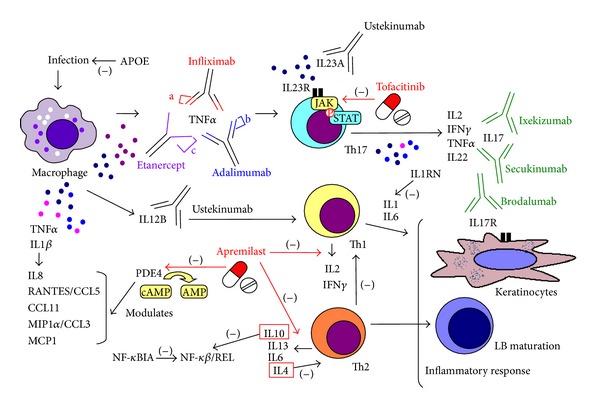
Simplified representation of the main mediators of inflammation in psoriasis, the therapeutic targets of biological drugs, and oral alternatives currently under development. Th: helper T lymphocyte; LB: lymphocyte B; APOE: apolipoprotein E; TNF: tumor necrosis factor; IL: interleukin; RANTES, chemokine regulated on activation normal T cells expressed and secreted; CCL: chemokine Cys-Cys motif ligand; MIP: macrophage inflammatory protein; MCP: monocyte chemoattractant protein; PDE4: phosphodiesterase 4; cAMP: cyclic adenosine monophosphate; IFN: interferon; JAK: Janus kinase; STAT: signal transducer and activator of transcription; NF-*κ*BIA: nuclear factor of kappa light polypeptide gene enhancer in B cells inhibitor; NF-*κ*B/REL: nuclear factor kappa B/v-rel reticuloendotheliosis viral oncogene complex; a: infliximab, mouse variable region; b: adalimumab, human variable region; c: etanercept, Human TNRFp75 (TNRF2); (−) indicates inhibition and (→) indicates stimulation.

T helper 17 (Th17) lymphocytes release IL22 and IL17 (Figure 1), which are highly expressed in psoriatic skin [18]. These lymphocytes also produce IL2, IFN*γ*, and TNF*α* (Figure 1) [3]. The proinflammatory cytokine TNF*α* plays a key role in the pathogenesis of psoriasis [19, 20]. Polymorphisms in the *TNF*α** gene may alter the release of this cytokine in healthy subjects [21]. A study performed in Caucasian patients with early-onset psoriasis showed a strong association with *TNF*α** polymorphisms (rs1800629 and rs361525) (Table 1) [19]. In this sense, a meta-analysis of 18 published case-control studies showed that when the GA + AA genotype was compared with the GG genotype, the risk of psoriasis increased for rs361525 and decreased for rs1800629 in *TNF*α** gene (Table 1) [22]. Kaluza et al. (2000) observed a decrease in TNF*α* production in peripheral blood mononuclear cells (47 cases and 43 controls) stimulated with mitogens in psoriatic patients who were A allele carriers of rs361525 (*TNF*α** gene) compared to controls [23]. Moreover, the authors found an association between the A allele in rs361525 in the *TNF*α** gene and increased production of TNF*α* and early onset of psoriasis (Table 1) [24]. A study performed in an Egyptian population (46 cases and 96 controls) revealed an association between SNPs in *TNF*α** (GG allele in rs1800629) and psoriasis (*P* < 0.05) (Table 1) [25]. However, no significant differences were found in rs1800629 and rs361525 in this gene in Korean patients with psoriasis (*n* = 103) and controls (*n* = 125) [26].

Reich et al. (1999) analyzed rs361525 and rs1800629 in *TNF*α** gene in patients with type I psoriasis (onset before 40 years; *n* = 100) and type II psoriasis (onset beyond 40 years; *n* = 51) and in healthy controls (*n* = 123) (Table 1) [27]. The results showed that the rs361525*A allele was more frequent and the rs1800629*A allele was less frequent in patients with type I psoriasis than in controls (*P* = 0.0012 and *P* = 0.041, resp.), although no differences were found between these polymorphisms and type II psoriasis [27]. Nedoszytko et al. (2007) analyzed 166 patients with psoriasis (134 with type I and 32 with type II) and 65 healthy controls [28] and found similar results to those of Reich et al. [27], with a higher prevalence of the A allele in rs361525 and lower frequency of the A allele in rs1800629 (*TNF*α** gene) in Caucasian patients than in controls (Table 1) [28]. A previous study performed in 99 Caucasian patients (64 with type I psoriasis and 35 with type II psoriasis) showed decreased frequency of the GG genotype and increased frequency of the GA genotype of rs361525 (*TNF*α** gene) in patients with type I psoriasis compared with controls (*n* = 123) (Table 1) [29]. Therefore, the GG genotype in this SNP is associated with a lower risk of type I disease [29].

The inflammatory response in psoriasis is characterized by production of TNF*α*, as seen above, and production of IL1*β* (Figure 1) [24]. In fact, this proinflammatory cytokine is overexpressed in psoriatic lesions [30]. An *in vitro* study in peripheral blood mononuclear cells (231 cases and 345 controls) revealed an association between the CC genotype in rs16944 in the *IL1*β** gene with increased production of IL1RA in response to lipopolysaccharide and IL10 and late-onset psoriasis (over 40 years) (Table 1) [24]. Johansen et al. (2010) observed that expression of IL1*β* was decreased 4 days after treatment with adalimumab (a human monoclonal antibody against TNF*α*) [30].

IL23 regulates and stimulates the activation, differentiation, and survival of Th17 lymphocytes (Figure 1) [31, 32] and is highly expressed in psoriatic lesions [18]. IL12 induces the production of IFN*γ* by Th1 (Figure 1) [33]. The p40 subunit of IL23 and IL12 is the therapeutic target of ustekinumab, a highly effective biological drug, thus suggesting that *IL12* and *IL23* play an important role in psoriasis [33–35]. Polymorphisms in *IL23R* and *IL12B* have been associated with susceptibility to psoriasis in both Caucasian [36, 37] and Asian patients [38, 39].

In Caucasians, a GWAS (1446 cases and 1432 controls) showed the combination of rs3212227 and rs6887695 in *IL12B* as a risk haplotype in psoriasis (Table 1) [37]. The authors also found an association between rs11209026 in the *IL23R* gene and psoriasis [37]. Capon et al. (2007) performed a study of 318 cases and 288 controls and found significant differences between the groups for rs3212227 in *IL12B* (*P* = 0.036) (Table 1) [40]. A subsequent GWAS with 1810 cases and 2522 controls found an association between SNPs in *IL23R* (rs7530511 and rs11209026) and *IL12B* (rs6887695 and rs3212227) and predisposition to psoriasis in Caucasian patients (Table 1) [36]. Smith et al. (2008) found similar results, associating these four SNPs with psoriasis [41], and Liu et al. (2008) identified an association between psoriasis and *IL23R* (rs11209026) and *IL12B* (rs6887695) (Table 1) [42]. Hüffmeier et al. (2009) analyzed the same four SNPs in 1114 patients and found a strong association between rs11209026 (*IL23R*) and rs3212227 (*IL12B*) and psoriasis (Table 1) [43]. Another recent study also associated rs11209026 in *IL23R* gene with psoriasis (Table 1) [2]. Other *IL12B* and *IL23R* susceptibility loci identified in GWAS in Caucasian patients include rs2201841 and rs2066808 (*IL23R*) and rs2082412 and rs2546890 (*IL12B*) (Table 1) [44, 45].

The SNPs rs11209026 in *IL23R* gene and rs3212227 in *IL12B* gene have also been studied in Japanese patients (143 cases and 100 controls), and the A allele (rs3212227) was more frequent in patients with psoriasis than in healthy subjects (Table 1) [46]. In a GWAS performed in a Thai cohort (206 cases and 144 controls), a marginally significant association was found between rs7530511 (*IL23R* gene) and psoriasis (Table 1) [38]; rs3212227 (*IL23R*) was also associated with the disease [38]. However, the authors did not find an association with rs6887695 in *IL12* gene [38]. A GWAS performed in a Chinese population (217 cases and 288 controls) identified other polymorphisms associated with psoriasis in *IL23R* (A allele rs11465817-A allele rs1343152 haplotype) and *IL12B* (rs6887695) (Table 1). The SNP in *IL12B* was replicated with 578 cases and 1422 controls, and the authors found a positive association with psoriasis [39].

Nair et al. (2009) found strong associations between psoriasis and other genes: *IL13*, which is involved in Th2 lymphocyte modulation (rs20541); TNF*α* interacting protein 3 (*TNFAIP3*) (rs610604, rs6920220, rs10499194, and rs5029939 [47, 48]) and TNFAIP3 interacting protein (*TNIP1*), which regulate the activity of nuclear factor kappa B (NF-*κ*B) [33]; *IL1RN*, which inhibits the activity of IL1; and *HLA-C* (rs12191877), which is involved in inflammatory responses [44] (Table 1). In addition, rs610604 (*TNFAIP3*) and rs17728338 (*TNIP1*), but not rs2066808 (*IL23R*) and rs397211 (*IL1RN*), were associated with psoriasis in a case-control study (Table 1) [2].

Ellinghaus et al. (2010) studied the TNF receptor-associated factor 3 interacting protein gene (*TRAF3IP2*) and identified an association between 2 SNPs and psoriasis (rs13210247 and rs33980500) (Table 1) [45]. This association was confirmed by Hüffmeier et al. (2010) in 2040 German patients with psoriasis vulgaris [49]. *TRAF3IP2* encodes a protein that interacts with NF-*κ*B/REL (v-rel reticuloendotheliosis viral oncogen) complexes and modulates IL17 pathways [45]. In another GWAS, rs240993 (*TRAF3IP2* gene) was associated with psoriasis in Caucasian patients (Table 1) [50]. In the GWAS performed by Ellinghaus et al. (2010), also in Caucasian patients, an association was identified between rs12191877 (*HLA-C*) and rs2145623 (nuclear factor of kappa light polypeptide gene enhancer in B cells inhibitor gene, NF-*κ*BIA) and psoriasis (Table 1) [45]. Feng et al. (2009) performed a GWAS (1359 cases and 1400 controls) and showed rs12191877 (*HLA-C*) to be a high-risk SNP for psoriasis (Table 1) [51]. The SNP rs8016947 in NF-*κ*BIA was associated with psoriasis (GWAS) (Table 1) [50].

Ellinghaus et al. identified new susceptibility loci [45], such as rs4649203 in *IL28RA* and rs12720356 in the tyrosine kinase 2 gene (*TYK2*) (Table 1) [50]. These authors also found an interaction between *HLA-C* and the endoplasmic reticulum aminopeptidase gene (*ERAP1*) (rs27524) [50]. In a Chinese population, another SNP in *ERAP1* (rs151823) was associated with early-onset psoriasis (less than 40 years) (GWAS, 8312 cases and 12919 controls) (Table 1) [52]. In a case-control study performed in patients with psoriasis (*n* = 1050; controls *n* = 1497), the SNPs rs8016947 (NF-*κ*BIA), rs4649203 (*IL28RA*), rs12720356 (*TYR2*), and rs27524 (*ERAP1*) were not associated with the disease [2].

Activation of Th1 lymphocytes was associated with the production of cytokines such as IL2 and INF*γ* [3, 18] (Figure 1). In a Korean population (114 patients and 281 controls), the rs2069762 (G allele) in *IL2* conferred a risk of psoriasis, mainly in the late-onset group (Table 1) [53]. As for *INF*γ**, rs2430561 has been associated with susceptibility to psoriasis (78 cases versus 74 controls) (Table 1) [54]. Furthermore, production of IFN*γ* was increased by DEFB4 (defensin beta 4A), a microbiocidal and cytotoxic peptide [55]. A significant association was found between rs2740091 and rs2737532 in *DEFB4* and predisposition to psoriasis in Caucasian patients (498 cases and 577 controls) (Table 1) [56]. IL18 also stimulates IFN*γ* production [57], and the presence of polymorphisms in the *IL18* gene (rs187238) was associated with susceptibility to psoriasis in Japanese patients (Table 1) [58].

Th2 lymphocytes release IL4, IL6, IL10, and IL13 [3] (Figure 1). A study performed in 114 psoriasis patients and 281 controls from Korea showed that rs2069762 (G allele) in *IL2* conferred a risk of developing the disease, mainly in late-onset psoriasis (Table 1) [53]. Moreover, the cytokines IL6 and IL10 seem to be important in the development of psoriasis [59]. In an Egyptian population (46 cases and 96 controls), an association was established between psoriasis and SNPs in *IL6* (CC genotype in rs1800795) and *IL10* (GG genotype in rs1800896) (Table 1) [25]. In addition, Craven et al. (2001) found differences in rs1800896 (*IL10*) genotype frequencies between patients with late-onset disease (*n* = 84) and controls (Table 1) [60]. However, results for the associations between rs1800896 in *IL10* gene and psoriasis are controversial, since several studies did not find any differences between cases and controls for this SNP [27, 59]. IL13 is involved in the differentiation and maturation of B cells and differentiation and function of Th17 lymphocytes [33]. Julia et al. (2012) found an association between rs20541 in *IL13* and psoriasis (Table 1) [2]. Moreover, the CCG haplotype of rs1800925-rs20541-rs848 in *IL13* was associated with susceptibility to psoriasis in a study performed in 1446 cases and 1432 controls (Table 1) [61]. In contrast, Duffin et al. (2009) found these associations with psoriatic arthritis, but not with psoriasis [62], and other authors found that rs20541 and rs1800925 in *IL13* gene were involved in psoriatic arthritis but not in psoriasis [63].

Other cytokines and chemokines associated with psoriasis include IL19, IL20, IL15, and MCP1 (monocyte chemoattractant protein). Minor alleles of rs2243188 and rs2243158 in *IL19* have a protective effect in patients with the disease (Table 1) [64]. In a case-control study (340 cases and 199 controls), the G allele in rs1713239 (*IL20*) was associated with psoriasis in a Chinese population (Table 1) [65]. Kingo et al. (2004) found an association between G allele carriers of rs2981572 (*IL20*) and predisposition to psoriasis in Caucasian patients (Table 1) [66]. Polymorphisms in the IL20 receptor (*IL20RA*) have also been associated with psoriasis (Table 1) [67]. Of note, the haplotype in *IL19* and *IL20* exhibited a susceptibility factor for the development of psoriasis [68]. IL15 induces the activation of the Janus kinase/signal transducer transcription activation factor (JAK/STAT) pathway and may trigger an immune response in psoriatic lesions [57, 69]. Polymorphisms in *IL15* (rs2857261, rs10519613, and rs1057972) have been associated with psoriasis in a Chinese population (Table 1) [69]. However, in a Caucasian population, no clear association was found between rs1057972 and rs10519613 in *IL15* gene and psoriasis [70]. 

MCP1 is a CC-type chemokine that plays a role in the recruitment of monocytes and T lymphocytes in inflammation [71]. Wang et al. (2008) found high serum levels of MCP1 in patients with psoriasis compared with controls [71]. The SNP rs10224611 (GG or AG genotype) in the *MCP1* gene may confer susceptibility to psoriasis (507 cases and 530 controls) (Table 1) [71].

Other genes associated with psoriasis include signal transducer and activator of transcription 4 (*STAT4*), apoliprotein E (*APOE*), vitamin D receptor (*VDR*), and cytotoxic T lymphocyte-associated protein 4 (*CTLA4*). Zervou et al. (2009) found a weak association between the T allele in rs7574865 (*STAT4*) and predisposition to psoriasis (Table 1) [72]. *APOE* may play a role in psoriasis by modifying the proliferation of mitogen-activated T lymphocytes and ensuring protection against some infections (Figure 1) [73]. Other authors have reported the APOE-*ε*4 allele to be a risk factor for the development of severe form of psoriasis [74]. In addition, 2 SNPs in the *APOE* gene (rs429358 and rs7412) have been associated with chronic plaque psoriasis and guttate psoriasis (Table 1) [75].

Several authors have demonstrated the role of *VDR* in the pathogenesis of psoriasis [76, 77]. Rucevic et al. (2009) described possible effects of *VDR* polymorphisms on the immune system, namely, immunomodulation, stimulation of cellular differentiation, and inhibition of proliferation [78]. The TaqI polymorphism (allele T) in *VDR* was associated with familial psoriasis in a Turkish population [79]. In addition, the A allele in rs451635 (*VDR* gene) was protective against susceptibility to nonfamilial psoriasis (Table 1) [76]. In contrast, Zuel-Fakkar et al. (2011) did not find any association between the polymorphisms ApaI and TaqI in *VDR* and psoriasis [77]. 

CTLA4 is a protein that downregulates activation of T lymphocytes [80]. The GG haplotype of rs3087243-rs231775 in *CTLA4* has been associated with psoriasis, but the analysis of these SNPs individually revealed no statistically significant associations (Table 1) [81]. Thus, in other studies, rs231775 in *CTLA4* gene was not associated with the disease in Korean [82] or Caucasian [80] populations.

Moreover, in a recent review the authors have emphasized other SNPs in genes associated with psoriasis (Table 1) [83]: interferon induced with helicase C domain 1 (*IFIH1*; rs17716942), late cornified envelope (*LCE*; rs4085613, rs4845454, rs1886734, rs4112788, rs6701216, and rs4112788), and ring finger protein 114 (*RNF114*; rs2235617 and rs495337). These genes have also been related with immune system (Table 1): *IFIHI* with response to viral infections, *LCE* with epidermal skin barrier function, and *RNF114* with T-cell activation. Although, the SNP rs67011216 in *LCE* gene has been associated with psoriasis in a GWAS study of 223 patients with psoriasis (91 of them with psoriatic arthritis) [42], other authors did not find this association in patients with psoriatic arthritis (*n* = 1057 cases and *n* = 5575 controls) [84]. Previously, Zhang et al. (2009) have found an association between rs4112788 in *LCE* gene and psoriasis in a GWAS performed in Chinese population [85]. A case-control study performed in patients with psoriatic arthritis has found this same association in Caucasian population [86].

In addition, Hébert et al. (2012) supported that the knowledge of risk genes for psoriasis may be useful to predict the response to treatment in patients with this disease [83].

In summary, the literature on the genes involved in immune system that participate in the pathogenesis of psoriasis indicates that *IL23R, IL10, TNF*α*, IL12B, GBP6, IL6, IL13, TNFAIP3, TNIP1, IL1RN, HLA-C, NF-*κ*BIA, APOE, VDR, IFN*γ*, IL2, IL4, IL15, TNFRSF1B, MCP1, CTLA4, DEFB4, STAT4, IL18, IL19, IL20, IL20RA, ERAP1, IL1B, TRAF3IP2, IL28RA*, *TYK2*, *IFIH1*, *LCE*, and *ZNF313* play an important role in the development of this disease.

## 3. Pharmacogenetics of Biological Drugs

### 3.1. Biological Drugs

The use of agents that block the action of TNF*α* (infliximab, etanercept, and adalimumab) has shown clear benefits in the treatment of patients with inflammatory diseases such as psoriasis [87]. TNF*α* induces the production of proinflammatory cytokines such as IL1 and IL6 (Figure 1), which in turn limits leukocyte migration and expression of adhesion molecules by endothelial cells and leukocytes. Neutralization of the biological activity of TNF*α* leads to an overall reduction in inflammation. Although anti-TNF*α* therapy is safe and well tolerated, some adverse events have been reported [88].

Advances in knowledge of the metabolic pathways involved in the pathogenesis of psoriasis and related diseases have led to the search for new therapeutic targets and the development of new biological drugs [10]. Such is the case of ustekinumab, a novel human immunoglobulin IgG1*κ* monoclonal antibody that binds strongly to the p40 subunit shared by IL12 and IL23 (Figure 1). This drug was designed to block the inflammatory cascade of Th1 and Th17 lymphocytes, since the altered behavior of keratinocytes in psoriasis probably results in deregulation of these pathways (Figure 1) [89]. In general, ustekinumab was well tolerated [90].

As mentioned above, psoriasis is mediated by the Th1/Th17 response. New biological therapies—both anti-IL17 agents (ixekizumab and secukinumab) [91, 92] and anti-IL17R agents (brodalumab) [93]—are being developed for the treatment of moderate-to-severe plaque psoriasis (Figure 1). Anti-IL17 drugs are now in phase III trials and may become new alternatives to ustekinumab and anti-TNF therapy [9]. Findings for anti-IL17 and anti-IL17R drugs illustrate the importance of the role of IL17 in the pathogenesis of psoriasis [18, 94].

### 3.2. Other Treatments of Psoriasis in the Future

Biological drugs are well tolerated and improve the PASI-75 (Psoriasis Area and Severity Index reduction ≥75%) score at week 12 [88, 92, 93, 95, 96]. Their main disadvantages are that injectable administration may cause rejection in some patients. Orally administered alternatives—tofacitinib and apremilast—are being developed (Figure 1).

Tofacitinib is a small JAK1/3 inhibitor molecule that was developed to treat psoriasis and other inflammatory diseases (Figure 1) [97]. The JAK family plays a key role in signal transduction from cytokine receptor in lymphocytes to STAT, which is involved in immune responses (Figure 1) [10, 98]. 

Apremilast is a PDE4 inhibitor that increases levels of cyclic adenosine monophosphate (cAMP) (Figure 1), which activates the protein kinase A and modulates the cytokines involved in the immune response of psoriasis (decreases TNF*α*, IL23, and IFN*γ* and increases IL10) [3]. PDE4 inhibitors cause anti-inflammatory activities [99], such as modulation of the synthesis and release of cytokines and chemokines from immune system cells. Stimulation with TNF*α* and IL1*β* can release several mediators: IL8, eotaxin-1, macrophage inflammatory protein 1-*α* (MIP1*α*/CCL3), MCP1, and chemokine regulated on activation, normal T cells expressed and secreted (RANTES/CCL5) (Figure 1) [99]. PDE4 inhibitors also suppress the production of inflammatory mediators by Th1 (IL2, IFN*γ*), Th2 (IL4), and macrophages (TNF*α*) but increase IL10 synthesis (Figure 1) [99]. Phase II studies have shown an acceptable tolerability and safety profile [100]. Phase III clinical trials of apremilast are ongoing.

Below, we review a selection of pharmacogenetics studies evaluating the efficacy and safety profile of biological drugs.

### 3.3. Pharmacogenetics

Only two studies have reported the effect of polymorphisms on the response to drugs used to treat psoriasis. In the first, Tejasvi et al. (2012) evaluated associations between two SNPs in *TNFAIP3* (rs2230926 and rs610604) and the response to TNF therapy in a cohort from Michigan (*n* = 433 patients) and a cohort from Toronto (*n* = 199 patients), both comprising patients with psoriasis and psoriatic arthritis [15]. The SNP rs610604 in *TNFAIP3* gene had previously been associated with predisposition to psoriasis and psoriatic arthritis [101]. The authors showed a favorable response to anti-TNF drugs (etanercept, infliximab, and adalimumab) and etanercept in carriers of the G allele of rs610604 in *TNFAIP3* in their Michigan cohort (OR = 1.5 and OR = 1.64, resp.) (Table 1). The T-G haplotype of rs2230926-rs610604 (*TNFAIP3*) was also associated with the response to anti-TNF in this cohort (Table 1). The authors did not find significant differences between rs610604 in *TNFAIP3* gene and adalimumab or infliximab analyzed individually or between the SNPs studied and the response to anti-TNF drugs in the Toronto cohort. The study presented the differences in the results between the two cohorts, stating that the reduced size of the Toronto cohort was a limitation of the study [15]. 

The other study was performed in 80 Greek psoriatic patients (43 women and 37 men) treated with adalimumab, infliximab, and etanercept. The authors analyzed five polymorphisms in three genes: *TNF*α** (rs361525, rs1800629, rs1799724), *TNFRSF1A* (rs767455), and *TNFRSF1B* (rs1061622) [14]. Genotyping data revealed an association with response to treatment after 6 months; the patients who achieved a reduction in the PASI score >75% were classified as responders and those with a reduction of ≤50% were classified as nonresponders [14].

Vasilopoulos et al. [14] found an association between a polymorphism in *TNF*α** (CC genotype for rs1799724; *P* = 0.027) and in *TNFRSF1B* (TT genotype for rs1061622; *P* = 0.019) and a better response to anti-TNF treatment (Table 1). The statistical analysis of each agent separately revealed an association between these genotypes and a positive response to etanercept after 6 months of therapy (*P* = 0.002 and *P* = 0.001, resp.). However, these SNPs were not associated with a good response to infliximab or adalimumab. The authors explained these differences by the mode of action of biological drugs (etanercept binds to soluble TNF*α*, and adalimumab and infliximab bind to transmembrane TNF*α*). The tests of association between the haplotype rs1799724-rs1061622 (*TNF*α**-*TNFRSF1B* genes) and the response to anti-TNF drugs showed significant differences (*P* < 0.05) for CT, CG, and TG. It is important to note that Vasilopoulos et al. excluded rs361525 (*TNF*α**), rs1800629 (*TNF*α**), and rs767455 (*TNFRSF1A*) from the statistical analysis because of a deviation from the Hardy-Weinberg equilibrium [14]. Nevertheless, other authors have reported that a deviation in Hardy-Weinberg equilibrium indicates a real association between genotype and disease [102]. 

Before treatment of psoriasis can be personalized, more studies should investigate the polymorphisms presented in this review, as well as other polymorphisms and their possible association with drugs used in the treatment of psoriasis. One recent review reported a role for SNPs in psoriasis-related autoimmune diseases (psoriatic arthritis, rheumatoid arthritis, and Crohn's disease) that could play a role in the response to anti-TNF drugs [8]. 

## 4. Conclusions

Our review focused only on those polymorphisms associated with the immune system and psoriasis. Current knowledge is limited, and many other SNPs not associated with immune system may be implicated in the development of psoriasis. Larger studies are necessary to obtain a better understanding of this complex disease, the pathways involved in its pathogenesis, and its pharmacogenetic implications in order to develop more effective and safer drugs that can be administered on a personalized basis.

## Figures and Tables

**Table 1:** Single-nucleotide polymorphisms (SNPs) in genes associated with psoriasis.

Gene	Role in immune system*	SNP	MAF**	Minor allele	Population	References
*IL23R *	Encodes a subunit of the receptor required for IL23A signaling. This protein associates constitutively with JAK2 and binds to transcription activator STAT3	rs7530511	0.125	T	Caucasian, Japanese, Chinese	[33, 34, 36–38, 41]
		rs2201841	0.275	C	Caucasian	[44, 45]
		rs11209026	0.067	A	Caucasian	[2, 33, 34, 36, 37, 41–43]
		rs11465817	0.279	A	Chinese	[39]
		rs1343152	0.357	C	Chinese	[39]
		rs2066808	0.092	C	Caucasian	[44]

*IL10 *	Encodes a cytokine produced by monocytes and lymphocytes that downregulates the expression of Th1 cytokines and blocks NF-*κ*B activity. It enhances B-cell survival, proliferation, and antibody production and regulates the JAK-STAT signaling pathway	rs1800896	0.467	A	Caucasian, Egyptian	[25, 60]

*TNF*α**	Encodes a proinflammatory cytokine produced by macrophages. TNF*α* is implicated in multiple roles such as cell proliferation, differentiation, and apoptosis	rs1800629	0.217	A	Caucasian, Egyptian, Korean	[19–22, 25–29]
		rs361525	0.131	A	Caucasian	[19, 20, 22–24, 26–29]
		rs1799724	0.158	A	Caucasian	[14]^#^

*IL12B *	IL12B is a cytokine expressed by activated macrophages that serves as an essential inducer of Th1 cell development	rs6887695	0.217	T	Caucasian, Chinese	[33, 36, 37, 39, 41, 42]
		rs3212227	0.225	C	Caucasian, Japanese, Chinese	[33, 36–38, 40–43, 46, 103]
		rs2082412	0.225	A	Caucasian	[44]
		rs2546890	0.438	G	Caucasian	[45]

*GBP6 *	Interferon induces GBP that hydrolyzes GTP to both GDP and GMP	rs928655	0.288	G	Caucasian	[42]

*IL6 *	Encodes a cytokine that induces inflammatory responses through IL6R*α* and maturation of B cells	rs1800795	0.467	G	Egyptian	[25]

*IL13 *	Encodes a cytokine produced by activated Th2 that is involved in maturation and differentiation of B cells. IL13 downregulates macrophage activity and inhibits the production of proinflammatory cytokines and chemokines	rs20541	0.233	T	Caucasian	[2, 44, 61]
		rs848	0.242	T	Caucasian	[61]
		rs1800925	0.196	T	Caucasian	[61]

*TNFAIP3 *	TNF induces the expression of TNFAIP3, which inhibits NF-*κ*B activation and TNF-mediated apoptosis. TNFAIP3 is involved in cytokine-mediated immune and inflammatory responses	rs610604	0.408	C	Caucasian	[2, 15, 44]^#^
		rs6920220	0.175	A	Caucasian	[33, 44, 47]
		rs10499194	0.175	T	Caucasian	[33, 44, 47]
		rs5029939	0.042	G	Caucasian	[44, 47, 48]
		rs2230926	0.027	G	Caucasian	[15]^#^

*TNIP1 *	Encodes TNFAIP3 interacting protein 1, which plays a role in the regulation of NF-*κ*B activation	rs17728338	0.075	A	Caucasian	[2, 44]

*IL1RN *	IL1RN inhibits IL1 and modulates immune and inflammatory responses	rs397211	0.164	G	Caucasian	[44]

*HLA-C *	HLA class I molecules play a central role in the immune system by presenting peptides derived from endoplasmic reticulum lumen	rs12191877	0.125	T	Caucasian	[44, 45, 51]
		rs10484554	0.135	T	Caucasian, Chinese	[2, 42, 104]
		rs1265181	0.258	C	Chinese	[35, 104]
		rs3134792	0.111	G	Caucasian	[105]

*NF-*κ*BIA*	Encodes a member of the NF-*κ*B inhibitor family, which interacts with REL dimers to inhibit NF-*κ*B/REL complexes, which are involved in inflammatory responses	rs2145623	0.290	C	Caucasian	[45]
		rs8016947	0.465	T	Caucasian	[50]

*APOE *	APOE plays a role in the proliferation of T lymphocytes and protects against some infections in patients with psoriasis [73]	rs429358	0.078	APOE*4	Caucasian	[75]
		rs7412	—	—	Caucasian	[75]

*VDR *	Encodes the nuclear hormone receptor for vitamin D3, which regulates immune response pathways	rs4516035	0.381	C	Caucasian	[76]

*IFN*γ**	Encodes a soluble cytokine with antiviral, immunoregulatory, and antitumor properties, and it is a potent activator of macrophages	rs2430561	**—**	**—**	Caucasian	[54]

*IL2 *	Encodes a cytokine that is important for the proliferation of T and B lymphocytes	rs2069762	**—**	**—**	Korean	[53]

*IL4 *	IL4 is a pleiotropic cytokine involved in the modulation of Th2 immune responses. IL4 receptor also binds to IL13, which may contribute to many overlapping functions of this cytokine and IL13	rs2243250	0.137	T	Korean	[53]

*IL15 *	Encodes a cytokine that regulates T-cell and natural killer activation and proliferation. *IL15* also induces the activation of JAK kinases, as well as the phosphorylation and activation of STAT3, STAT5, and STAT6	rs2857261	0.431	G	Chinese	[69]
		rs10519613	0.102	A	Chinese	[69]
		rs1057972	—	—	Chinese	[69]

*TNFRSF1B *	TNFRSF1B is a TNF*α* receptor that mediates the recruitment of antiapoptotic proteins	rs1061622	0.239	G	Caucasian, Japanese	[14]^#^

*MCP1 *	*MCP1* encodes a cytokine characterized by two cysteines separated by a single amino acid that displays chemotactic activity for monocytes and basophils	rs1024611	0.305	G	Caucasian	[71]

*CTLA4 *	Encodes a protein which inhibits T cells	rs3087243	0.460	A	Caucasian	[81]^##^
		rs231775	0.389	G	Caucasian	[81]^##^

*DEFB4 *	DEFB4 is a member of a family of microbicidal and cytotoxic peptides made by neutrophils	rs2740091	—	—	Caucasian	[56]
		rs2737532	—	—	Caucasian	[56]

*STAT4 *	In response to cytokines, the STAT proteins are phosphorylated and translocate to the cell nucleus, where they act as transcription activators. STAT transduces IL12, IL23, and IFN type I signals in T lymphocytes and regulates the differentiation of Th cells	rs7574865	0.230	T	Caucasian	[72]

*IL18 *	IL18 stimulates production of IFN*γ* in Th1	rs187238	—	—	Japanese	[58]

*IL19 *	IL19 is a member of the IL10 cytokine subfamily with a role in inflammatory responses	rs2243188	0.230	A	Caucasian	[64, 68]
		rs2243158	0.085	C	Caucasian	[64]

*IL20 *	Encodes a cytokine structurally related to IL10 and transduces its signal through STAT3 in keratinocytes	rs1713239	0.177	G	Chinese	[65]
		rs2981572	—	—	Caucasian	[64, 66, 68]

*IL20RA *	Encodes a receptor for IL20, a cytokine that may be involved in epidermal function	rs1342642	0.314	A	Caucasian	[67]
		rs1184860	—	—	Caucasian	[67]
		rs1167846	0.246	T	Caucasian	[67]
		rs1167849	0.285	A	Caucasian	[67]

*ERAP1 *	Encodes an aminopeptidase involved in trimming HLA class I-binding precursors so that they can be presented on HLA class I	rs151823	0.093	A	Chinese	[52]
		rs27524	0.332	A	Caucasian	[50]

*IL1B *	Encodes a cytokine produced by activated macrophages which plays an important role in the inflammatory response	rs16944	0.358	A	Caucasian	[24]

*TRAF3IP2 *	Encodes a protein that interacts with TRAF proteins and plays a central role in innate immunity in response to pathogens, inflammatory signals, and stress	rs13210247	0.080	G	Caucasian	[45, 49]
		rs33980500	—	—	Caucasian	[45, 49]
		rs13196377	0.053	A	Caucasian	[49]
		rs13190932	0.058	A	Caucasian	[49]
		rs240993	0.250	T	Caucasian	[50]

*IL28RA *	Encodes a receptor complex that interacts with IL28A, IL28B, and IL29. The expression of these cytokines can be induced by viral infection	rs4649203	0.239	G	Caucasian	[50]

*TYK2 *	Encodes a member of the JAK protein family that promulgate cytokine signals by phosphorylating receptor subunits. TYK2 is a component of IFN I and II signaling pathways and may play a role in antiviral immunity	rs12720356	0.124	C	Caucasian	[50]

*IFIH1 *	Encodes a protein that mediates induction of IFN response to viral RNA [83]	rs17716942	0.195	C	Caucasian	[50]

*LCE *	Encodes a protein that plays a role in skin barrier function [83]	rs4085613	0.403	T	Caucasian	[50]
		rs4845454	0.403	C	Caucasian	[50]
		rs1886734	0.407	A	Caucasian	[50]
		rs4112788	0.403	A	Caucasian	[50]
		rs6701216	0.137	T	Caucasian	[42]
		rs4112788	0.417	T	Chinese	[85]

*ZNF313 *	Encodes a protein that is involved in T-cell activation [83]	rs2235617	0.432	G	Caucasian	[50]
		rs495337	0.430	A	Caucasian	[105]

*Data from NCBI web page [57]; **MAF: minor allele frequency for Caucasian population (data from HapMap web page [106] and Alfred [107]). IL: interleukin; R: receptor; JAK: Janus kinase; STAT: signal transducer and activator of transcription; Th1: type 1 helper T lymphocyte; TNF: tumor necrosis factor; GBP: guanylate-binding protein; GTP: guanosine triphosphate; GDP: guanosine diphosphate; GMP: guanosine monophosphate; TNFAIP: TNF-alpha interacting protein; TNIP1: TNFAIP3 interacting protein; IL1RN: interleukin 1 receptor antagonist; HLA: human leukocyte antigen; NF-*κ*BIA: nuclear factor of kappa light polypeptide gene enhancer in B cells inhibitor, alpha; REL: v-rel reticuloendotheliosis viral oncogene; APOE: apolipoprotein E; VDR: vitamin D receptor; TNFRSF1: tumor necrosis factor receptor superfamily; MCP: monocyte chemoattractant protein; CTLA4: cytotoxic T lymphocyte-associated protein 4; DEFB4: defensin beta 4A; IFN: interferon; ERAP: endoplasmic reticulum aminopeptidase; TRAF3IP: TRAF3 (TNF receptor-associated factor 3) interacting protein; IRAK: interleukin-1 receptor-associated kinase; TYK: tyrosine kinase; IFIH1: interferon induced with helicase C domain 1; LCE: late cornified envelope; RNF114: ring finger protein 114; ^#^association between psoriasis and response to anti-TNF treatment; ^##^haplotype GG of rs3087243-rs231775 associated with psoriasis.
